# Elevated Cerebral Spinal Fluid Cytokine Levels in Boys with Cerebral Adrenoleukodystrophy Correlates with MRI Severity

**DOI:** 10.1371/journal.pone.0032218

**Published:** 2012-02-16

**Authors:** Troy C. Lund, Paul S. Stadem, Angela Panoskaltsis-Mortari, Gerald Raymond, Weston P. Miller, Jakub Tolar, Paul J. Orchard

**Affiliations:** 1 Division of Pediatric Blood and Marrow Transplant, University of Minnesota, Minneapolis, Minnesota, United States of America; 2 Department of Neurogenetics, Kennedy Krieger Institute, Baltimore, Maryland, United States of America; Hôpital Robert Debré, France

## Abstract

**Background:**

X-linked adrenoleukodystrophy (ALD) is a metabolic, peroxisomal disease that results from a mutation in the *ABCD1* gene. The most severe course of ALD progression is the cerebral inflammatory and demyelinating form of the disease, cALD. To date there is very little information on the cytokine mediators in the cerebral spinal fluid (CSF) of these boys.

**Methodology/Principal Findings:**

Measurement of 23 different cytokines was performed on CSF and serum of boys with cerebral ALD and patients without ALD. Significant elevations in CSF IL-8 (29.3±2.2 vs 12.8±1.1 pg/ml, p = 0.0001), IL-1ra (166±30 vs 8.6±6.5 pg/ml, p = 0.005), MCP-1 (610±47 vs 328±34 pg/ml, p = 0.002), and MIP-1b (14.2±1.3 vs 2.0±1.4 pg/ml, p<0.0001) were found in boys with cALD versus the control group. The only serum cytokine showing an elevation in the ALD group was SDF-1 (2124±155 vs 1175±125 pg/ml, p = 0.0001). The CSF cytokines of IL-8 and MCP-1b correlated with the Loes MRI severity score (p = 0.04 and p = 0.008 respectively), as well as the serum SDF-1 level (p = 0.002). Finally, CSF total protein was also significantly elevated in boys with cALD and correlated with both IL-8, MCP-1b (p = 0.0001 for both), as well as Loes MRI severity score (p = 0.0007).

**Conclusions/Significance:**

IL-8, IL-1ra, MCP-1, MIP-1b and CSF total protein were significantly elevated in patients with cALD; IL-8, MCP-1b, and CSF total protein levels correlated with disease severity determined by MRI. This is the largest report of CSF cytokine levels in cALD to date, and identification of these key cytokines will provide further insight into disease progression and perhaps lead to improved targeted therapies.

## Introduction

Adrenoleukodystrophy (ALD) is a peroxisomal disease affecting the nervous system, adrenal cortex, and testis resulting from a mutation in the *ABCD1* gene in humans with a disease prevalence of approximately 1 in 20,000 [1], [2]. The *ABCD1* gene is located on the X-chromosome and it encodes the peroxisomal protein ATP-binding cassette-transporter D1 responsible for the trafficking of very-long-chain fatty acids (VLCFA), and over 460 different mutations have been reported [3]. ALD can display several different phenotypes; these phenotypes include cerebral childhood adrenoleukodystrophy (cALD), a cerebral juvenile form, a cerebral adult form, adrenomyloneuropathy (AMN), and isolated Addison's disease. Currently, there is no known link between mutation and phenotypes, making it difficult to predict the possible progression of the disease for each individual [1]. In cALD, the 5-year survival rate after first appearance of the clinical onset of cerebral inflammation is 59% [3].

Current treatments for cALD are sparse. Although a low-fat diet to reduce VLCFA intake coupled with a lipid supplement known as Lorenzo's Oil has shown to normalize VLCFA concentrations in the blood and perhaps delay the onset of cALD, these treatments have not demonstrated an effect on the progression of cerebral inflammation once it has started [4], [5]. The only treatment that has shown to slow the progression of the cerebral inflammation and demyelination once the clinical onset of cerebral inflammation has been observed is hematopoietic stem cell transplant (HSCT) [2], [5]. In studies determining the effectiveness of HSCT, a clear distinction in the long term survival of the patients was observed between those in the early-stages of cerebral inflammation versus those with a higher amount of cerebral inflammation as determined by MRI [2]. Therefore, the earlier identification of boys who will develop cALD is needed, and we need to better understand the mediators of neuroinflammation in cALD in order to determine those that would benefit most from HSCT.

Although cALD is thought to be driven by an infiltrative neuroinflammatory process, there has not been a comprehensive analysis of potential inflammatory mediators involved with cALD [6]. Previous work measuring inflammatory cytokines including tumor necrosis factor-alpha, interleukin-1 beta, interleukin-4, interleukin-6 and interferon-gamma in brain lesions in postmortem samples from patients with cALD showed that levels cytokine mRNA were lower than that compared to samples take from patients with multiple sclerosis and surprisingly *more similar* to expression levels in control white matter [7]. Additionally, there have been mixed data on whether TNF-alpha plays a role in the pathogenesis of cALD, as protein levels have not been found not to be different in cALD versus control specimens, although there is some suggestion that there is increased bioactivity of TNF-alpha in the serum of boys with cALD [8], [9].

We have collected a large number of serum and cerebral spinal fluid (CSF) samples from boys with cALD prior to undergoing hematopoietic stem cell transplant and performed a comprehensive analysis of multiple of cytokines using a Luminex-based inflammatory cytokine panel. Identification of the key mediators will help us better understand the neuroinflammatory process and perhaps give clues to new targeted therapies.

## Methods

CSF and serum samples were evaluated using the 22-plex, human panel A, (R&D Systems, Minneapolis, MN) measured with the Luminex system (Luminex, Austin, TX) analyzed by Bioplex software (BioRad, Hercules, CA). This panel is summarized in Table 1 and includes: ENA-78, bFGF, G-CSF, GM-CSF, IFN-gamma, IL-1alpha, IL-1beta, IL-1ra, IL-2, IL-4, IL-5, IL-6, IL-8, IL-10, IL-17, MCP-1, MIP-1alpha, MIP-1beta, RANTES, TNF-alpha, TPO, and VEGF. SDF-1alpha was measured by sandwich ELISA (R&D Systems). All values were interpolated from standard curves generated with the relevant recombinant human proteins provided with the commercial kits. Total protein level was determined by the hospital acute care lab and extracted from the medical chart.

**Table 1:** List of inflammatory factors evaluated in CSF and serum samples.

Cytokine Name	Abbreviation
Epithelial derived neutrophil activating peptide 78 or CXCL5	ENA-78
Basic fibroblast growth factor	bFGF
Granulocyte colony stimulating factor	G-CSF
Granulocyte macrophage colony stimulating factor	GM-CSF
Interferon gamma	IFN-gamma
Interleukin 1alpha	IL-1alpha
Interleukin 2beta	IL-1beta
Interleukin 1 receptor antagonist	IL-1ra
Interleukin 2	IL-2
Interleukin 4	IL-4
Interleukin 5	IL-5
Interleukin 6	IL-6
Interleukin 8	IL-8
Interleukin 10	IL-10
Interleukin 17	IL-17
Monocyte chemotactic protein 1 or CCL2	MCP-1
Macrophage inflammatory protein 1alpha or CCL3	MIP-1a
Macrophage inflammatory protein 1beta or CCL4	MIP-1b
Regulated upon activation, Normal T-cell expressed or CCL5	RANTES
Tumor necrosis factor alpha	TNF-alpha
Thrombopoietin	TPO
Vascular endothelial growth factor	VEGF
Stromal derived factor 1alpha	SDF-1alpha

### Objectives

To determine which inflammatory cytokines were elevated in the CSF and plasma of boys with cALD. To determine if inflammatory cytokine elevation correlated with disease severity shown by MRI.

### Participants

Patients with cALD (*n* = 36, median age 8.3 years) had CSF sampling done 2 to 6 months prior to hematopoietic stem cell transplant at the University of Minnesota. Control patients (*n* = 22, median age of 7.4 years for serum and *n* = 25, median age 6.8 years for serum) were those undergoing intrathecal methotrexate chemotherapy for a prior diagnosis of acute lymphoblastic leukemia and were at least 3 months into maintenance therapy and without CSF leukemia. The CSF was withdrawn prior to administration of methotrexate. Unavailability of “healthy” controls and risk of attaining CSF from healthy children established these patients as the most appropriate control group available and has been previously published [10].

### Description of Procedures or Investigations undertaken

Boys with cALD were referred to the University of Minnesota Division of Pediatric Blood and Marrow Transplant as candidates for hematopoietic stem cell transplantation. They all had an initial MRI prior to transplant and required sedation. During the sedated MRI, a lumbar puncture was performed and 3 milliliters of CSF was obtained and analyzed for protein level, cell count, and cytokine analysis. The same procure was used to collect the “control” samples except, sedation is not typically done for children in ALL maintenance therapy. Serum was obtained at the same time as the CSF.

### Ethics

This study and the use of all bodily fluids was approved by the Committee on the Use of Human Subjects in Research at the University of Minnesota. Informed written consent was obtained for all patient samples from the parents or guardians on behalf of the child participants. Patient written assent was also obtained if patients were greater than 8 years of age.

### Statistical methods (if applicable)

Each sample was run in duplicate and an average value calculated to arrive at the cytokine concentration using standard curves generated with the relevant recombinant human proteins provided with the commercial kits. Means for the cALD and control groups were calculated and subjected to a two-tailed Student's t-test to compute a p-value. A linear regression analysis from cytokines of interest and MRI Loes score was performed using Prism software (version 5.0b).

## Results

**Figure 1:**
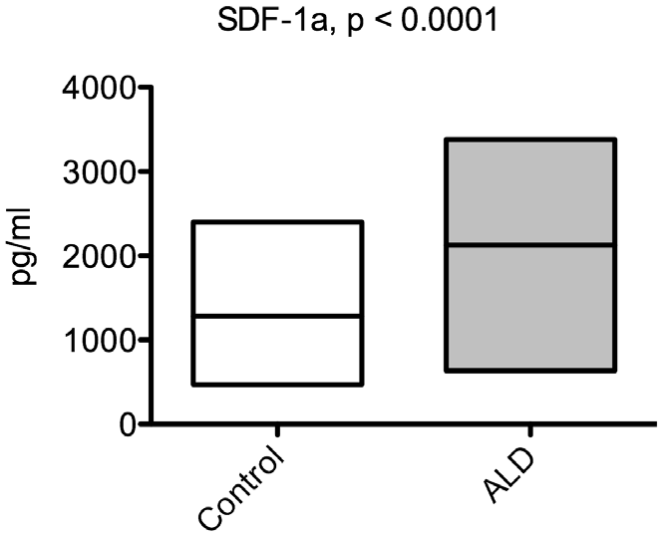
Serum SDF-1 is elevated in boys with cerebral ALD. SDF-1 levels were determined by ELISA. Boxes show min and max, and the bar is at the mean. A Student's unpaired t-test calculated p-value shown.

**Figure 2:**
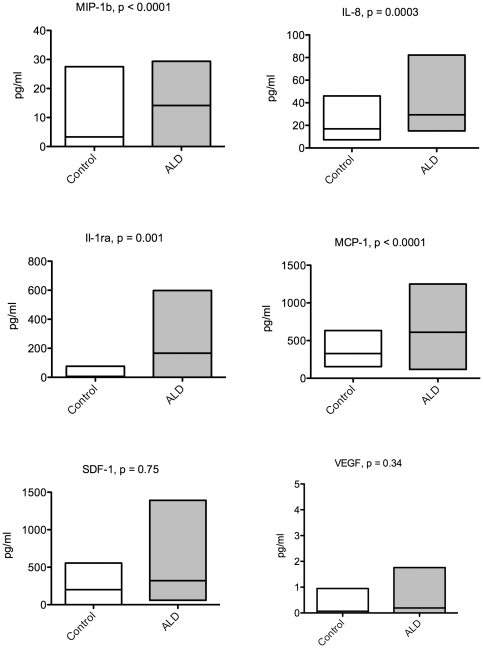
CSF inflammatory cytokines are elevated in boys with cerebral ALD. CSF cytokines were evaluated using a Lumunix system and SDF-1 by ELISA. Boxes show min and max, and the bar is at the mean. A Student's unpaired t-test calculated p-values shown.

We performed a comprehensive evaluation of inflammatory cytokine levels in the CSF and serum of boys with cALD summarized in Table 1. Our analysis of serum from control (n = 25, median age 6.8) versus cALD patients (n = 36, median age 8.3) showed no significant differences a significant differences except in the case when SDF-1 was analyzed with 1175±124pg/ml versus 2124±155 pg/ml, respectively, as shown in Figure 1 (p = 0.0001). None of the other cytokines in our analysis showed a significant difference between cALD and control patient serum (data not shown). Contrary to the serum, Figure 2 shows SDF-1 levels in the CSF were not different between cALD and controls with 321±62.3 pg/ml and 274±32.6 pg/ml (p = 0.54).

Further cytokine analyses performed on the CSF revealed higher levels in cALD samples compared to controls for IL-8 (29.3±2.2 vs 12.8±1.1 pg/ml, p = 0.0001), IL-1ra (166±30 vs 8.6±6.5 pg/ml, p = 0.005), MCP-1 (610±47 vs 328±34 pg/ml, p = 0.002), and MIP-1b (14.2±1.3 vs 2.0±1.4 pg/ml, p<0.0001) as shown in Figure 2. We also found VEGF to be elevated in the CSF of several of the boys with cALD and very few of the control patients, but because of the inconsistency in which it appeared, the means are not statistically significant.

**Figure 3:**
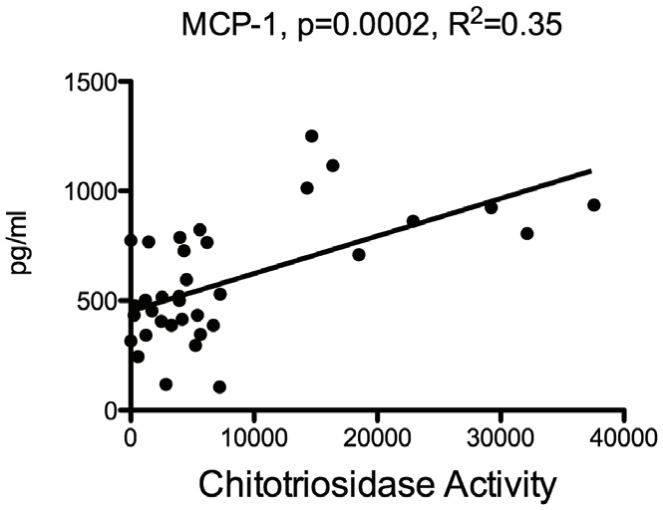
Correlation of CSF MCP-1 levels and chitotriosidase activity. Regression analysis was performed using CSF MCP-1 levels and CSF chitotriosidase activity as described by Orchard et al [10]. Statistical analysis was performed using Prism software.

We have recently shown that another marker, chitotriosidase is elevated in both the serum and the blood in boys with cALD [10]. The serum SDF-1 levels did not correlate with chitotriosidase levels (data not shown), but of the CSF cytokines, MCP-1 levels did correlate very well with chitotriosidase activity (p = 0.0002) (Figure 3), leading us to believe that these two inflammatory proteins could be linked by a pathophysiologic mechanism.

**Figure 4:**
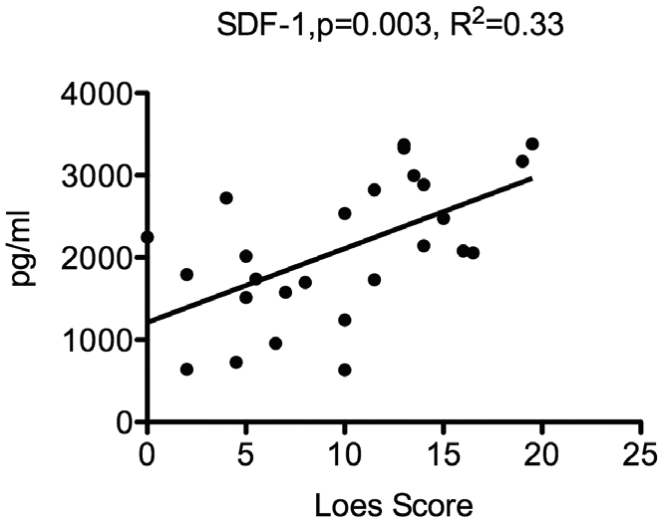
Correlation of serum SDF-1 levels and Loes MRI severity score. Regression analysis was performed using serum SDF-1 levels and each patient's pretransplant Loes score using Prism software.

**Figure 5:**
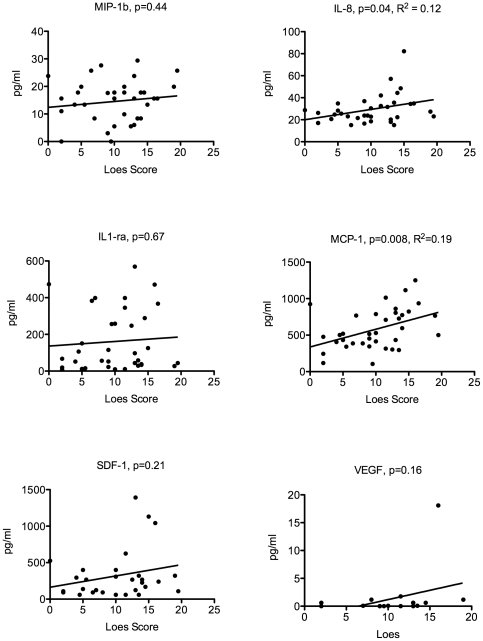
Correlation of CSF inflammatory cytokine levels and Loes MRI severity score. Regression analysis was performed using the previously determined CSF cytokine levels and each patient's pretransplant Loes score using Prism software.

The Loes Score is an MRI-based quantification of the amount of cerebral involvement based on a point system derived from location, extent of disease, and the presence of focal and/or global atrophy that is present in boys with cALD [11]. All boys referred to our center have an MRI prior to transplant that is scored per the scale described by Loes et al. Figure 4 shows that serum SDF-1 levels correlate significantly with the pretransplant Loes score (p = 0.003). In evaluation of the CSF cytokines that were elevated in the CSF of boys with cALD, we found that IL-8 and MCP-1 had a significant correlation with the pretransplant Loes score (p = 0.04 and p = 0.008 respectively), while the other cytokines did not correlate significantly shown in Figure 5.

**Figure 6:**
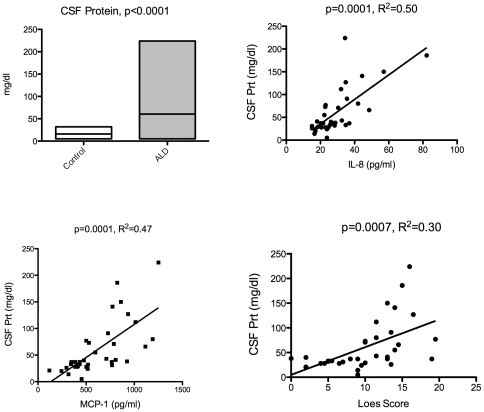
Elevated levels of CSF total protein correlate to IL-8, MCP-1, and Loes MRI severity score. CSF total protein levels were taken from the patients' medical record. Boxes show min and max, and the bar is at the mean. Regression analysis was performed using the previously determined CSF cytokine levels for IL-8 and MCP-1 as well as each patient's pretransplant Loes score using Prism software.

In a prior report, our institution characterized the CSF total protein in 25 boys with cALD [12]. Abnormally high values of CSF total protein were found in a majority of those tested, particularly from those with more advanced disease (as seen on MRI). There was no relationship between CSF IgG levels and IQ, but there was an inverse relationship between CSF myelin basic protein and IQ. In this current dataset, we again found markedly high levels of CSF protein (versus controls) as shown in Figure 6 (p<0.0001). And, in fact, the cytokines IL-8 and MCP-1 correlated with elevated CSF protein (Figure 6) although MIP-1b did not (data not shown). Finally, we also verified that CSF protein concentration correlated with Loes score as shown in Figure 6 (p = 0.0007, R^2^ = 0.30). These data suggest that the total protein level by itself can correlate with disease severity/inflammation, which although intuitive, has not been shown quantitatively.

## Discussion

In this study, we determined that there were significant elevations in inflammatory cytokine levels in the serum (SDF-1) and CSF (IL-8, IL-1ra, MCP-1, MIP-1b, and VEGF). The serum SDF-1and CSF IL-8 and MCP-1 correlated significantly with the pretransplant MRI Loes score severity and the CSF MCP-1 levels correlated significantly with chitotriosidase activity. Inflammation contributes to neuronal death in many inflammatory neurodegenerative disease such as Alzheimer's disease, multiple sclerosis, Parkinson's disease, and amyotrophic lateral sclerosis [13]. All of the identified cytokines that were elevated in cALD patients have been implicated to play a role in the regulation of an inflammatory response that is mediated both by the cells of the immune system and glial cells. In addition, the fact that TNF-alpha and IL-1 were not found to be different in our cALD samples correlates with prior work by McGuiness *et al* and Philips *et al* as they also found no difference in TNF-alpha protein levels in the CSF or serum from patients with cALD, although McGuiness did find there was increased TNF-alpha *bioactivity* in patients with cALD [8], [12].

This is the first report of the elevation of SDF-1 in the serum of patients with cALD. While SDF1 is typically thought of a homing factor for many cell types [14], it has been shown to be elevated in patients with various inflammatory diseases such as lupus, multiple sclerosis, and tick-borne encephalitis [15]–[17]. In boys with cALD, we hypothesize that it may reflect a level of chronic inflammation this exists in these boys. Why SDF1 is only elevated in the serum and not significantly higher in the CSF, while the opposite is true for the other cytokines we evaluated, is not known. Perhaps SDF-1 is being secreted by damaged cells in an unappreciated niche responding to VLCFA toxicity, which in the case of ALD could be the adrenal gland, but this, thus far, remains speculative.

MIP-1b (CCL4), IL-8 (CXCL8), and MCP-1 (CCL2) have all been identified as pro-inflammatory chemokines and can induce the migration of leukocytes to a desired site and have also been implicated in other inflammatory diseases such as multiple sclerosis, Alzheimer's disease, and HIV [18], [19]. MIP-1b is a chemoattractant for several immune response mediator cells including natural killer cells and monocytes, and MIP-1b mRNA has been shown to be increased in the inflammatory areas of brains affect by cALD [3]. In mouse models, IL-8 plays a role in leukocyte activation and chemotaxis and has been shown to provoke polymorphonuclear leukocyte recruitment that is correlated with the breakdown of the blood-brain barrier [18]. We speculate that IL-8 mediated destruction of the blood-brain-barrier may explain, in part, the findings of gadolinium enhancement (a sign of inflammation) seen on the MRI of boys with cALD. MCP-1 recruits monocytes via chemotaxis to sites of inflammation and has been identified to play a possible role in the other aforementioned neurodegenerative diseases [19].

Our finding that MCP-1 levels correlate with chitotriosidase activity is intriguing as chitotriosidase is a chitolytic enzyme produced by activated monocytes/macrophages and has been used as a biomarker in other storage diseases [20], [21]. Historically, the limited data that exists suggests that cALD is characterized by predominately a lymphocytic infiltrate rather than monocytic. With an increase in both chitotriosidase and MCP-1 (which can be produced by glial cells as well [22]), this may reflect monocyte/macrophage activation which has been shown in other inflammatory conditions [21], [23]. Alternatively, chitotriosidase could be originating from microglial cells which are known to be in an activated, and even apoptotic, state in cALD [23], [24]. Is unlikely that any cells in the CSF are producing chitotriosidase or any of the other cytokines studied here as the lumbar almost always zero white blood cells present (data not shown).

An antagonist of the biologic activity of IL-1, IL-1ra is elevated in many other conditions such inflammatory arthritis (reviewed by Arend [25]), so our finding of its elevation in the setting of cALD is not surprising. Whether it plays a key role in the pathogenesis of cALD or is serving only as a marker is unknown and worthy of further exploration.

We were able to show that serum SDF-1 and CSF IL-8 and MCP-1 significantly correlated with disease severity as determined by MRI. This seems logical that more damage seen on MRI would be associated with higher cytokine levels. Because of the large range of values in the paitients with cALD, they are not likely to be used as biomarkers alone. And the above analysis does not allow for conclusion about whether these cytokines *cause* the neuroinflammation or if they are elevated as a result of an untempered inflammatory response preceded by neuronal apoptosis initiated by VLCFA accumulation. These results, nonetheless, could show promise as early indicators for cerebral inflammation in ALD patients if measured prospectively. This is important because studies have shown that HSCT is significantly more effective if performed during the early stages of cerebral inflammation and the outcomes are improved [2], [5], [26], [27].

The finding of elevated CSF total protein levels in boys with cALD has been suggested in a prior study from our institution [12]. The fact that IL-8 and MCP-1 levels also correlated with CSF protein concentration is not surprising, but should not undermine the importance of the increased amounts of these cytokines because cells can respond to the local concentration gradient of these factors regardless of other protein content. Increased total protein in the CSF agrees with the inflammatory nature of cALD, and the correlation of total CSF protein with the Loes score is an interesting finding and confirms the prior report quantitatively. What is not known is the total makeup of the CSF protein. While CSF is known to contain immunoglobulin, myelin basic protein, as well as the cytokines described in this study, there are assuredly many other factors which may play a role in the pathophysiology of cALD which are currently being explored.

In this study, we have identified key cytokines that are found at elevated levels in the serum and CSF of boys with cALD in the largest cohort described to date. These factors could be possible therapeutic targets for future clinical interventions to halt the progression of the cALD. Finally, while dietary modifications or supplements may be attempted to prevent the onset of neurological symptoms, the only modality successful at slowing disease progression is HSCT. It is possible that the cytokines identified in this study may help, when combined with other factors, predict the clinical course post-HSCT in boys with cALD, as this can be difficult on an individual patient basis.

### Limitations

Ethically obtaining ideal control CSF samples was a challenge for this study. We used CSF from children who were receiving maintenance therapy for their acute lymphoblastic leukemia (and were in remission) as they were having routine spinal taps every 3 months. The main concern was possibly selecting children as control patients with suppressed CSF cytokines thereby skewing the data, but studies measuring cytokine levels in CSF of healthy children yielded very comparable results to our data, supporting that the cytokine levels in our control patients in this study are congruent with accepted values in the literature [28], [29].
